# The effect of consuming different dietary protein sources at breakfast upon self rated satiety, peptide YY, glucagon like peptide-1, and subsequent food intake in young and older adults

**DOI:** 10.1007/s00394-025-03839-y

**Published:** 2025-11-12

**Authors:** Anthony W. Watson, Anna Brooks, Lucy Moore, Sophie Barley, Adrian Holliday

**Affiliations:** 1 School of Biomedical, Nutritional and Sports Sciences, Newcastle University, Newcastle upon Tyne, UK; 2 Human Nutrition and Exercise Nutrition Centre, Newcastle University, Newcastle upon Tyne, UK

**Keywords:** Protein, Breakfast, Satiety, Plant protein, Animal Protein, Liquid breakfast

## Abstract

Interest in plant-based protein in the UK is increasing due to health, environmental, and ethical considerations. Recent studies have explored how different protein sources impact satiety and related gut hormone responses, with evidence suggesting varied responses between animal-based and plant-based proteins. Skewed protein intake patterns, especially at breakfast, present an opportunity for improving dietary protein distribution in populations who may require increased protein intake but often face appetite reductions. This study determined the acute effect of consuming a plant-based, high protein drink containing 30 g of protein (HPDp); an animal-based, high protein breakfast containing 30 g of protein (HPBa); and a low-protein (10 g), high-carbohydrate breakfast (HCLPB) on satiety hormone responses, subjective appetite and subsequent energy intake in older and younger populations when consumed at breakfast. Eighteen heathy adults completed this within-subject, counterbalanced, cross-over study, (12 under 35 years of age and six over 65 years of age). Measurements for appetite were obtained at baseline, 30, 60, 90, 120, 150, 180, 210 and 240 min, and plasma, GLP-1 and PYY at baseline, 30, 60, 90, 120, 180 min post breakfast consumption. No difference in appetitive responses was found between the HPDp and the energy- and protein-matched HPBa, with both eliciting greater GLP-1 and PYY (both *p* < 0.004) responses compared with a high carbohydrate, low protein meal. Subjective appetite was also suppressed to a greater extent with HPDp compared with HCLPB (*p* = 0.001). No differences were observed in *ad libitum* energy intake.

## Introduction

 The consumption of adequate quantities of quality protein is an essential part of a balanced diet. While dietary protein intake for good health remains a priority, focus is also placed on the environmental sustainability of protein. Alternative proteins is one of the key areas of focus of the Food and Agriculture Organisations’s (FAO) Programme Priority Area on Bioeconomy for Sustainable Food and Agriculture which aims to support the achievement of Sustainable Development Goal target 12.2—Sustainable management and efficient use of natural resources. This drive towards a shift from “conventional animal” protein sources to alternatives such as vegetive sources [57] has created space for products such as plant-based meat alternative foods and convenience foods such as plant-based, high protein drinks and snacks.

Protein source can determine the digestive and metabolic responses and may also be the case for appetitive responses. The satiating effect of proteins has long been researched and the general consensus is that when matched for energy, protein has a greater satiating effect than carbohydrate or fat [43]. This is likely mediated by the potency of amino acids to stimulate satiety peptides PYY [5] and GLP-1 [50], and to suppress the hunger hormone ghrelin [10, 12]. Focus has turned to the differing effects of protein sources upon satiety. Greater gut hormone and satiety responses have been observed after casein and pea protein ingestion compared with soy protein, but neither pea nor soy protein differed to whey protein [11], while others have shown whey to elicit greater gut hormone and satiety responses than both soy and casein proteins [64]. This indicates varying gut hormone responses within both animal and vegetative protein sources. Interestingly, Hawley et al. [29] showed that pea protein isolate may have a greater appetite-suppressing effect than whey in older, but not younger men, but this was independent of differences in gut hormone response. In terms of the effects of vegetive protein sources as part of a meal on appetite and satiety, there is conflicting evidence of attenuated GLP-1 response [45] and amplified PYY [35] response to energy- and protein-matched plant versus meat food products. A recent review by Del Bo’ et al. [24] indicates that plant-based meat alternatives are as satiating as animal-based comparators. However, outcomes again varied based on the protein source and the population under investigation.

Given that high protein products are consumed as food and drinks, the comparative appetitive effects of solid and liquid forms should be considered, and indeed the interactions between nutrient composition, protein source, and form. Evidence would point to weaker appetitive effects of drinks compared with food [2, 36, 44]. Nutrient-balanced liquid meal replacement drinks have been shown to elicit greater postprandial hunger and desire to eat than energy and nutrient-matched solid food snack bars [58, 61] in older adults, but with no difference in gut hormone response [61]. Similar attenuated hunger-suppression has been observed with high protein ingestion [39], suggesting that ingesting protein in liquid form reduces the satiety effect [36]. However, interactions between protein sources and form have not yet been determined.

In the UK the recommended daily allowance (RDA) of protein is 0.75 g kg BW^−1^ day^−1^ for all healthy adults from the age of 19 years. While most people achieve or exceed this recommended intake [7, 14], many seek a higher protein intake. A higher protein intake of 1.2–2 g kg BW^−1^ day^−1^ is recommended for regular exercisers to maximise recovery and adaptations to training [60], while the satiating effect of protein means high-protein diets can be effective for weight management strategies [17, 68]. There is also growing consensus in the literature through consortia such as the PROT-AGE group, that older adults require 1–1.2 g kg BW^−1^ day^−1^ [6]. Increasing or maintaining protein intake, a particularly satiating nutrient, may be a challenge in later life given that a reduction in appetite is experienced in ~ 30% of community dwelling older adults [18]. Indeed, recent evidence has highlighted that those with low appetite experience amplified gut hormone responses to a mixed meal [21, 31]. However, others have suggested that appetitive response to protein may change with age, weakening the satiety effect [16]. Nonetheless, this poses a paradoxical problem of increasing protein intake without compromising total energy intake and intake of other key nutrients.

The daily distribution of protein is skewed regardless of age. The lowest amount is commonly consumed in the morning [25], with high-carbohydrate meals often the preferred choice at breakfast [25]. This may make breakfast an opportunity to improve protein intake [28, 38]. There is, however, little understanding of how emerging plant-based products such as liquid meal replacements with substantial protein content (30 g+) impact satiety and subsequent food intake when consumed at breakfast and whether age influences these outcomes.

This study aims to understand the acute effect of consuming a plant-based, high protein drink (HPDp); an animal-based, high protein breakfast (HPBa); and a low-protein, high-carbohydrate breakfast (HCLPB) on circulating levels of total GLP-1, total PYY, subjective appetite measures and subsequent energy intake in older and younger populations when consumed at breakfast. It is hypothesised that plant-based protein, when consumed in liquid form, will induce weaker appetitive responses than an animal protein-based solid meal, but stronger responses than a commonly consumed low-protein, high-carbohydrate solid breakfast meal.

## Method

### Study design

The study followed a within-subjects design where participants completed three experimental conditions in a randomised, counter-balanced order: plant-based, high protein drink (HPDp); animal-based, high protein breakfast (HPBa); and low-protein, high-carbohydrate breakfast (HCLPB). Outcome measures were subjective appetite, lunch *ad libitum* energy intake, and circulating concentrations of the satiety gut hormones. The study adhered to the ethical guidelines as outlined in the Declaration of Helsinki. Ethical approval was granted by the Newcastle University Faculty of Medical Sciences Research Ethics Committee (Ethics number: 2414/24643).

### Participants

Twelve low-to-moderately active younger adults (aged 18–35 years) not living with obesity and a sub-group of six older adults (≥ 65 years) were recruited for this study. Participants were recruited through poster distribution at Newcastle University facilities and at institutions in the Newcastle and North Tyneside areas, such as libraries and community centres. Inclusion criteria were: aged 18–35 years for younger adult and ≥ 65 years for the older adult sub-group; low-to-moderate activity level, as classified by score of < 3000 MET mins week^− 1^ on the International Physical Activity Questionnaire (IPAQ) [20]; not living with obesity, classified by a body mass index (BMI) of < 30 kg m^− 2^ for YA and < 33 kg m^− 2^ for older adults [67]; not attempting to change bodyweight or body composition; not taking medication likely to impact on appetite; non-smoker; no known food allergies; free from metabolic disease.

### Enrolment and familiarisation

Participants attended the Human Nutrition Suite, School of Biomedical, Nutritional and Sports Sciences, Newcastle University for enrolment and familiarisation. Informed written consent was obtained after a thorough verbal and written explanation of the study. Height and weight were measured using a stadiometer (SECA 232 height measure) and digital scales (SECA 875 digital scales).Participants were screened for any known food allergies and habitual physical activity was assessed using the IPAQ.

Participants were then familiarised with the test meals to be consumed on the trial visit. A sample of the animal-based, high protein drink was provided to ensure palatability. Palatability of the other two breakfast meals was confirmed after describing the ingredients. Participants were provided a small portion (~ 150 g) of the lunch meal. They were asked to taste the meal to confirm palatability such that they would be happy to consume the food on experimental trials until “satisfyingly full”.

### Procedures

Participants arrived at the laboratory in a fasted state, between 08:00 and 09:00, to commence the experimental trial. All participants had consumed a standardised, nutrient-balanced evening meal of macaroni cheese, yoghurt, and orange juice (777 kcal; 48% energy from carbohydrate, 33% fat, 18% protein) a minimum of 10 h prior to arrival, and had abstained from exercise, caffeine, and alcohol on the day before the experimental visit (with adherence confirmed at the start of each trial). Upon waking, participants drank 300mL of water to ensure euhydration.

The experimental trial began with a measure of subjective appetite using the visual analogue scale (VAS) method. A cannula was then inserted into the antecubital vein of one arm. After a 10-minute rest period, a fasted, rested blood sample was obtained. Participants then consumed one of the three test breakfasts: HPDp, HPBa, or LPB. All breakfasts were consumed in between 8 and 10 min.

**Fig. 1:**
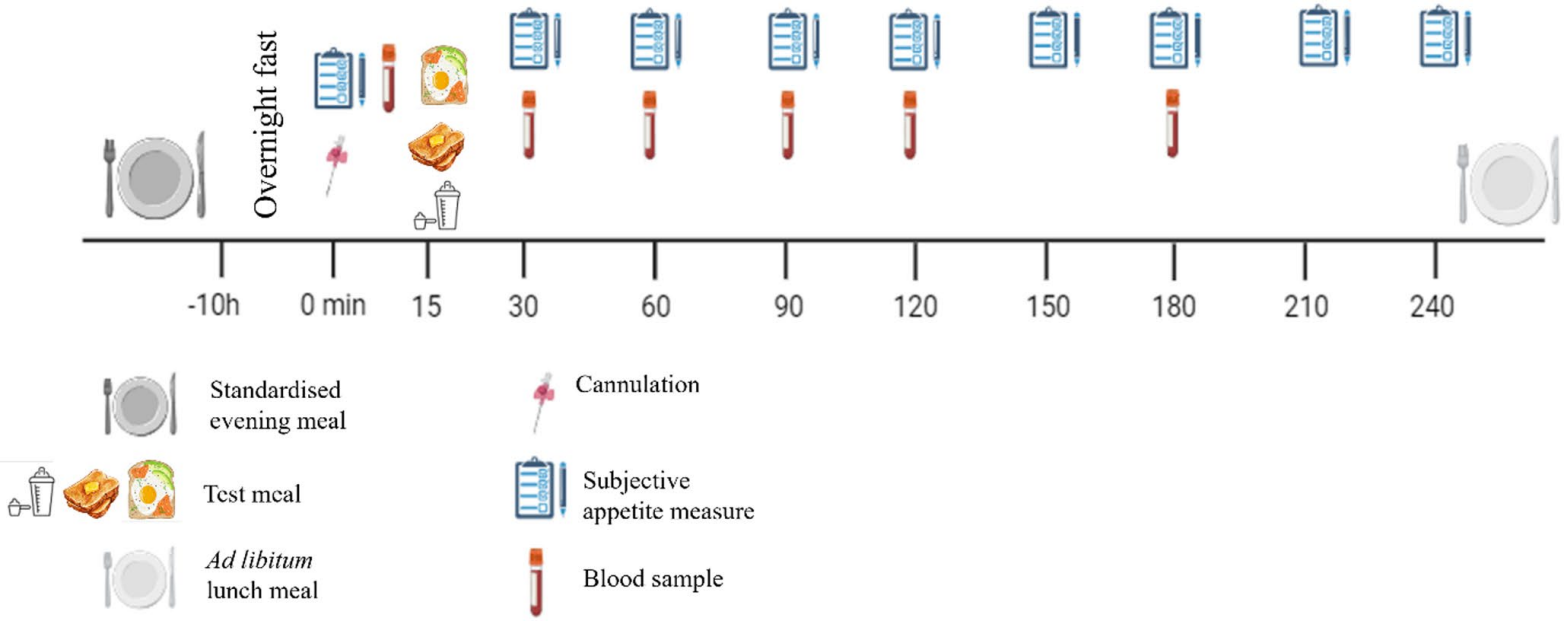
Schematic of study protocol

Upon consumption of breakfast and 30 min after the cannulation, subjective appetite was measured, and a second blood sample was obtained. Participants then rested for a further 210 min, with appetite measured every 30 min and blood samples obtained at 60, 90, 120, and 180 min (see Fig. 1). Participants remained seated during this period, being free to read, watch television or use a laptop computer. Activity was monitored by a member of the research team to minimise exposure to food cues in reading and viewing material.

After the final blood sample at 180 min, the cannula was removed. Participants were then presented with a nutrient-balanced *ad libitum* lunch meal of pasta, Bolognese sauce and grated cheese (energy density = 1.79 kcal g^− 1^. 50% energy from carbohydrate, 15% protein, 35% fat). Upon completion of the meal, the trial was complete.

### Test meals

The energy and macronutrient composition of all test meals are shown in Table 1. Meals were closely matched for energy content. The HPDp and HPBa were closely matched for total protein content. The HPDp consisted of 100 g of a commercially-available, high-protein and high-fibre meal replacement drink powder (Huel Powder vanilla flavour, Huel Ltd), constituted with 500mL of water. The drink was prepared and chilled at 4 ºC a minimum of 15 min before consumption. The HPBa meal consisted of two large boiled eggs (Sainsbury’s, free-range, large, 120 g), and salmon (Sainsbury’s salmon trimmings, 30 g) on a single slice of toast (Warburton’s Batch Seeded Loaf, 46 g) with margarine spread (Flora Light, 10 g) and chia seeds (5 g). The HCLPB consisted of two slices of toast (Kingsmill 50/50 bread, 94 g), with margarine (Flora Light, 24 g) and hazelnut chocolate spread (Nutella, 24 g).

**Table 1:** Energy and macronutrient composition of the HPDp, HPBa, and HCLPB meals

	HPDp	HPBa	HCLPB
Energy (kcal)	400	404	402
Protein (g)	30.0	29.8	10.3
Carbohydrate (g)	39.0	19.0	52.3
Fat (g)	12.0	22.0	15.7
Fibre (g)	7.8	4.5	5.2

### Outcome measures

#### Subjective appetite

Subjective appetite was measured using the 4-item VAS method, assessing hunger, fulness, desire to eat and expected consumption (Flint et al., 2000). Participants placed a vertical mark on an ungraded, 100 mm horizonal line anchored on both ends with extreme answers to the question posed (“How hungry are you?”, “How full are you?”, “How strong is your desire to eat?” and “How much would you expect to eat right now?”). The distance from the left-hand side anchor to the mark was measured to record a score. A single composite score was calculated from the four items as: (hunger score + (100-fullness score) + desire to eat score + expected intake score) / 4 [30].

#### *Ad libitum* food intake

Food intake at lunch was assessed using a homogeneous pasta-based *ad libitum* test meal [3, 27], with participants instructed to eat until “satisfyingly full.” As an empty bowl can promote the cessation of eating prior to satiation [66], the “bottomless bowl” method was adopted whereby each bowl of pasta was replaced with a fresh, full bowl before the bowl was emptied [23]. The meal was consumed in isolation and free from food cues and distractions (such as a mobile phone). No time limit was imposed on the meal. Each bowl was weighed before and after serving to calculate the mass consumed. The energy consumed was calculated from the known energy density of the meal (1.79 kcal g^− 1^).

#### Plasma concentration of PYY and GLP-1

Blood was collected in EDTA-treated blood collection tubes before being centrifuged at 2000 *g* and 4 ºC for 15 min to separate plasma from cellular material. Plasma was aliquoted and stored at −80 ºC for later analysis. PYY and GLP-1 concentrations in plasma were measured by enzyme-link immunosorbent assay (ELISA) using commercially available kits (Human PYY (total) ELISA kit, Merck Millipore; Multi Species GLP-1 Total ELISA kit, Merck Millipore). Sensitivity of PYY and GLP-1 kits were 1.4 pg mL^− 1^ and 1.5 ng mL^− 1^, respectively. Coefficients of variation were 3.2% and 6.4%.

### Statistical analyses

An a priori power calculation was conducted to determine the sample size required to provide adequate statistical power to detect a large effect (η^2^_p_ = 0.14) in subjective appetite based on previous studies determining meaningful changes in subjective appetite with breakfasts of different protein content and composition. With statistical power of 0.8 and an alpha value of 0.05, a sample of at least 12 participants was required to detect a large difference between treatments (*d* = 1.2).

All values are presented as mean ± SD (mean ± SEM in figures). To assess PYY and GLP-1 responses to each breakfast, change-from-baseline concentration was calculated. Mixed models were used to fit the change from baseline data for GLP1, PYY and self-reported hunger with treatment (HPDp, HPBa, HCLPB), and repetition of measure entered into the model as fixed effects along with the interactions between each of these i.e. treatment × timepoint and treatment × age group (under 35 and over 65 years). Baseline scores for the relevant variable were controlled as a covariate. Participant ID number was added to the model as a random effect. Significant fixed effects were analysed further using pairwise comparisons with Bonferroni corrections for multiple comparisons utilised on outcomes.

To determine associations between gut hormones, subjective appetite, and lunch *ad libitum* energy intake, both between- and within-subject correlation analyses were conducted, correcting for repeated-measures [8, 9]. In both instances, correlations were conducted using AUC for subjective appetite and GLP-1 and PYY concentrations across the trial period, calculated using the trapezoid method.

Outliers were identified from fasted appetite scores and hormone concentrations (> 1.5 × interquartile range above the third quartile or below the first quartile). Statistical significance was determined at an alpha level of 0.05. All statistical analyses were conducted using Statistical Package for Social Sciences (SPSS, Version 29.0.1.0).

## Results

### Participant characteristics

Participant characteristics can be found in Table 2.

**Table 2:** Participant characteristics

	Total	18–30	> 65
Age (years)	38.06 ± 23.77	21.33 ± 1.75	71.50 ± 3.40
Weight (kg)	67.96 ± 9.85	64.20 ± 8.43	75.48 ± 8.03
Height (m)	1.68 ± 8.95	1.68 ± 10.73	1.70 ± 2.29
BMI (kg/m^2^)	23.66 ± 3.11	22.60 ± 2.58	25.78 ± 2.79
IPAQ score (MET minutes per week)	404.10 ± 225.5	412.00 ± 189.0	396.00 ± 337
SNAQ score	28.00 ± 2.04	16.00 ± 1.41	12.00 ± 0.50

Outcomes displayed as mean ± standard deviations

### GLP1

**Fig. 2:**
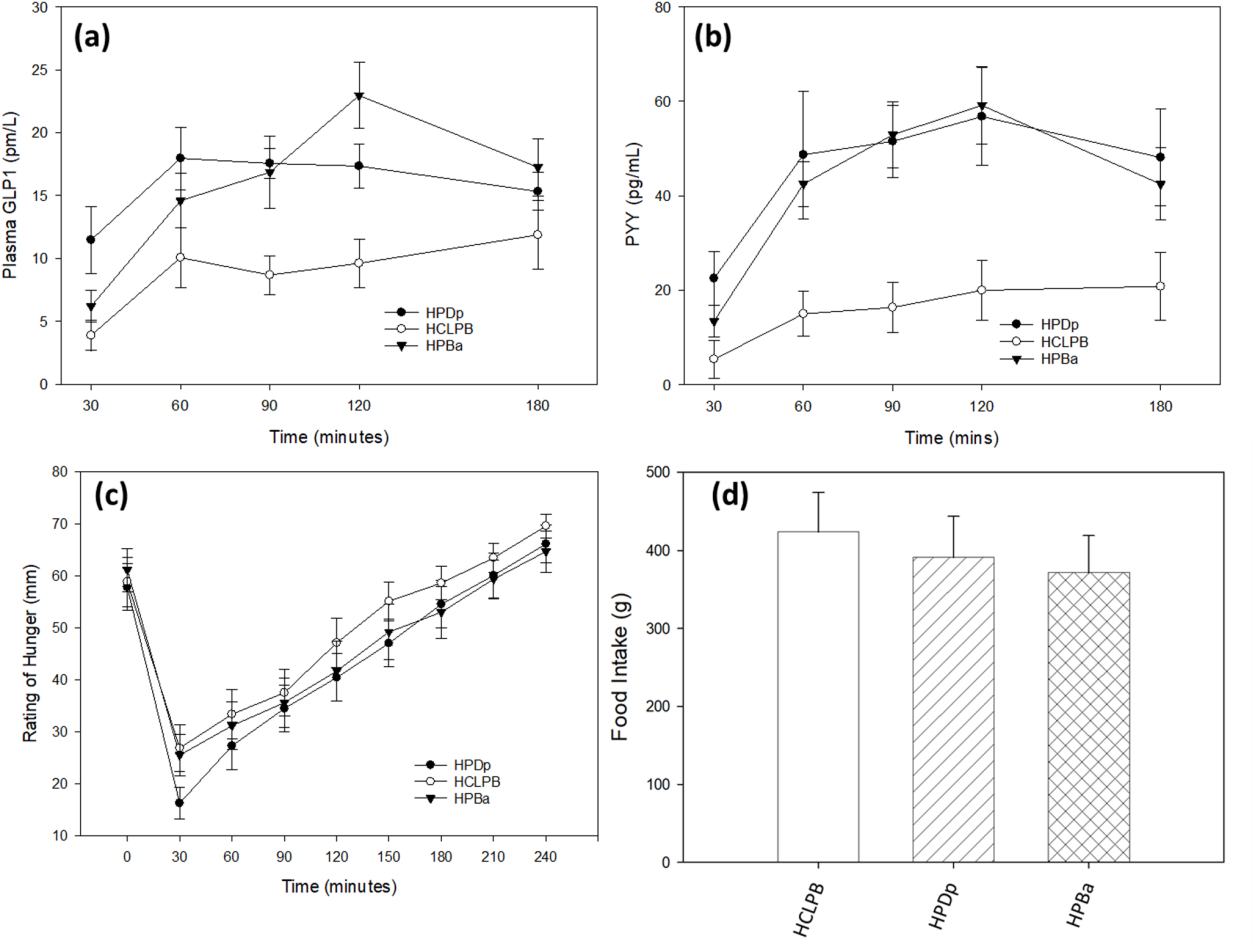
Mean ± SEM change from baseline plasma GLP-1 (pmol/L) (**a**); change from baseline plasma PYY (pg/mL) (**b**); food intake (g) during ad libitum meal (**c**); Self rated hunger (mm) (**d**)

There was a significant treatment effect for GLP-1 [F (2,77) = 9.05; *p* < 0.001, Fig. 2a]. Pairwise analysis showed a greater GLP-1 response after consumption of the HPDp (*p* < 0.001) and HPBa (*p* = 0.003) when compared with HCLPB. There was no difference between HPDa and HPBa (*p* > 0.999). There was no treatment × time interaction (*p* = 0.074). There was no treatment × age group interaction (*p* = 0. 393). See Fig. 2a.

### PYY

There was a significant effect of treatment for PYY [F (2,86) = 10.87 *p* < 0.001, Fig. 2b]. Pairwise analysis showed a significant increase in plasma PYY levels after consumption of the HPDp (*p* < 0.001) and HPBa (*p* = 0.003) when compared to HCLPB. There was no difference between HPDp and HPBa (*p* > 0.999). There was no significant treatment × time interaction (*p* = 0.252. No treatment × age group interaction was found (*p* = 0.486). See Fig. 2b.

### Subjective appetite

There was a significant effect of treatment [F(2,261) = 6.51 *p* = 0.002, Fig. 2c]. Pairwise analysis showed a lower appetite rating after consumption of the HPDp (*p* = 0.001) and a trend reduction after consumption of HPBa (*p* = 0.051) when compared with HCLPB. There was no difference between HPDp and HPBa (*p* = 0.899). There was no treatment × time interaction (*p* = 0.981) or treatment × age group interaction (*p* = 0.850). See Fig. 2c.

### Energy intake at *ad libitum* meal

There was no effect of treatment for energy consumed at the *ad libitum* meal [F(2,32) = 1.500. P*p* = 0.220. Figure 2d]. There was also no treatment × age group interaction. See Fig. 2d.

### Association between gut hormones, subjective appetite, and *ad libitum* energy intake

To assess the association between gut hormone concentration, subjective appetite, and *ad libitum* energy intake between participants (e.g., if those with higher GLP-1 concentration consumed less food), between-subject correlations were conducted. Subjective appetite AUC was strongly and significantly associated with *ad libitum* energy intake (*r* = 0.688, *p* = 0.002). AUC for both GLP-1 and PYY concentrations showed trends for significant, moderate-strength, positive associations with *ad libitum* energy intake (*r* = 0.431, *p* = 0.074 and *r* = 0.427, *p* = 0.078, respectively). There was no association between AUC for either GLP-1 or PYY concentration and AUC for subjective appetite (*r* < |0.2|, *p* > 0.1).

To assess the associations between a participant’s changes in gut hormones, changes in subjective appetite, and *ad libitum* energy intake (e.g., if a large increase in GLP-1 for a participant was associated with the consumption of less food), within-subject correlations were conducted. Neither subjective appetite, GLP-1 nor PYY AUC were associated with *ad libitum* energy intake (all *r* < |0.21|, *p* > 0.1). Neither GLP-1 nor PYY AUC were associated with subjective appetite AUC (both *r* < |0.18|, *p* > 0.1).

## Discussion

To our knowledge, this is the first study to explore the interaction between protein source and food form on appetitive responses. We show no difference in appetitive responses between a plant-based high protein drink and an energy- and protein-matched animal-based solid meal, with both eliciting greater satiety hormone responses compared with a high carbohydrate, low protein meal. Subjective appetite was also suppressed to a greater extent with HPDp compared with HCLPB. These responses were not age dependent.

Consumption of both HPDp and HPBa resulted in larger increases in plasma GLP-1 and PYY concentrations compared with HCLPB. These findings align with previous research indicating that protein intake can stimulate the secretion of GLP-1 an PYY at a greater extent than carbohydrates [5, 47, 50]. The lack of difference between plant- and animal-based high protein meals suggests that the source of protein may not significantly influence GLP-1 and PYY secretion when the foods are macronutrient and energy matched and consumed as part of a meal.

Visually, it appears the increase in satiety hormone concertation occurred more rapidly in the HPDp than the HPBa condition. This may be due to the quicker gastric emptying of a liquid meal [1, 42]. The fibre content of the HPDp meal was greater than that of the HPBa condition (7.8 g vs. 4.5 g). High fibre intakes can increase gastric distention and reduce the rate of gastric emptying [32], which may favour satiety [15, 32]; however, this does not appear to have been the case in the present study with this magnitude of difference in fibre content. Interestingly, it has been suggested that saponins—bioactive plant-based compounds—may induce satiety [13] via the stimulation of GLP-1 release [37]. This is another potential mechanism for the visually-observed more immediate satiety hormone response to the plant based protein meal, which deserves further investigation.

In addition to hormonal responses, we observed a significant effect of treatment on subjective appetite. Participants reported a significantly lower appetite in the HPDp condition, and there was a trend towards lower appetite in the HPBa condition, when compared with HCLPB. No significant difference was found between HPDp and HPBa. These results suggest that plant-based proteins, when consumed in a drink, are equally as effective at reducing appetite, compared with animal-based protein consumed as a solid meal. As well as determining the statistical significance of differences in appetite, the practical meaningfulness should be considered. A VAS-measured difference of ≥ 15 mm for appetite is deemed meaningful for likely impact on eating behaviour and energy intake [51]. At 30 min, the difference in composite appetite score between HPDp and HCLPB was 11 ± 14 mm, while the difference between HPDp and HCLPB was 9 ± 11 mm (which somewhat aligns with the visually-observed more immediate increase in satiety hormones in the HPDp condition). Differences between conditions did not exceed 10 mm at any other point. It is therefore likely that such differences between condition in subjective appetite are not practically meaningful with regards to influencing eating behaviour. Previous studies have reported greater satiety of soy-based meat replacement meals, compared with beef [45] and chicken meals [69]. It is possible that this greater satiety effect was not observed in the present study due to a difference source of plant-based protein (pea proteins vs. soy protein). It is also possible that a greater satiety effect of plant-based protein was negated by consumption in a less-satiating liquid form [2, 36, 44].

The likely non-meaningful differences in subjective appetite ratings between conditions, particularly beyond 30 min, was reflected in comparable energy consumption at the *ad libitum* lunch meal. While high protein preloads have typically been shown to reduce energy intake and a subsequent meal [12, 54], a higher protein intake at breakfast may have limited impact on ad libitum intake at lunch provided ~ 4 h later [52]. As such, our findings are perhaps unsurprising. However, it is worth noting that there was a non-significant but observable lower food consumption in the older group, with an approximate 160 kcal lower intake in the high protein conditions compared with HCLPB. This points towards sensitivity to the satiating effect of protein in the present group of older adults—which contradicts the findings of Clegg and Williams [16]—but with no difference between protein source or form.

The agreeable small-magnitude differences in subjective appetite between conditions and lack of difference in energy intake were observed despite enduring differences in gut hormone profiles between the two high protein conditions and the HCLPB condition. The association between fasting or pre-meal concentration of satiety hormones and energy intake is unclear. Infusion of GLP-1 [65] PYY [4], or both [59] typically shows anorexigenic effects; however, the relationship between hormone concentration and energy intake is not always observed with lower infusion rates [53, 56] or is weaker when the circulating concentration induced is closer to the physiological range [65]. Studies that have observed changes in satiety hormones in response to nutritional manipulation and then assessed *ad libitum* food intake have observed associations [12, 22, 26, 34] and no associations [12, 26, 34, 63] between hormone concentrations and energy intake. In the present study, subjective appetite was associated with *ad libitum* energy intake between-subjects. Somewhat unexpectedly and counterintuitively, there were moderate-strength, positive associations between both GLP-1 and PYY and *ad libitum* energy intake which approached statistical significance. This is despite their accepted anorexigenic effects. Within-subject, changes in subjective appetite, GLP-1, and PYY across the conditions were not associated with changes in *ad libitum* energy intake. This is perhaps not surprising given the lack of differences in energy intake across the conditions. Our data does, however, question the impact of changes in GLP-1 and PYY concentrations on subjective appetite and food intake in response to modest nutritional manipulation.

A sub- group of older adults were recruited, allowing for the determination of the effect of age on appetitive responses. There were no age effects for any of the outcome measures. It is acknowledged that the sample size of the older adult sub- group was small—the study was powered to detect differences between treatments and not specifically to detect differences between age groups—and hence reduces statistical power and increases the likelihood of type II error. Consequently, our findings must be interpreted with caution. However, as a preliminary exploration, our data would suggest that the observed effects of liquid form plant-based protein are not age dependent. It is, however, worth noting that in older adults, mean lunch energy intake was ~ 160 kcal greater after the high-carbohydrate and low-protein breakfast, compared with the two high-protein breakfasts. Previous studies have indicated that the satiety effect of protein is weaker in older adults (Clegg and Williams), while other have shown that high protein intakes at breakfast do not result in compensatory reductions in food intake at the next meal or across the day [19] in older adults. Our findings, however, do not point to a dampened satiety effect of protein in older adults, regardless of protein source and meal form.

The broad appeal of a high protein diet necessitates versatile options for those seeking to increase protein intake. Our data suggest that a plant-based, high-protein drink may be a viable alternative to the more conventional protein consumption of eating predominantly animal-based, high-protein foods. This is likely appealing to those with preference for plant-based foods and those seeking sustainable dietary sources of protein. A heavy reliance on animal-based protein products may have excluded those with such preferences from public health interventions and research; the adoption of a plant-based alternative such as that used in this study will likely favour inclusivity.

In addition, effective strategies for increasing protein intake in free-living interventions and studies should have low burden and align with dietary trends, such as a desire for “breakfast-on-the-go” [55]. Approaches should also aim to overcome common barriers to healthy eating. These include a lack of time [40, 49, 62], desire for convenience [48, 62], taste preferences [49, 62], and lack of motivation [33, 46], with additional barriers unique to older adult, such as limited cooking skills and physical impairment [41]. Liquid meal replacement drinks, reconstructed from powder, offer a time-effective, convenient, low-skill, and low-effort alternative to cooking solid meals. In the present study, the preparation time for the HPDp condition was two minutes (excluding chilling time), compared with approximately 12 min for the meal of the HPBa condition. While we did confirm tolerability of each condition at the point of enrolment, taste preference for each of the conditions was not assessed in the present study. Future studies should determine taste responses and acceptability of plant-based, liquid high-protein meals to assess likely effectiveness in free-living interventions.

A limitation of this study is that we did not include either a plant-based solid food or an animal-based liquid drink condition. This would have allowed for isolation of both factors—protein source and form—for comparison. However, this would have increased the levels of the dependent variable and the participant burden, so was deemed not appropriate. Instead, an ecologically-valid approach was adopted in order to assess differences between typical breakfast foods and a commercially-available plant-based meal replacement drink.

## Conclusion

In conclusion, our study demonstrates that breakfasts containing protein from plant and animal sources significantly increase plasma GLP-1 and PYY levels and reduce self-rated hunger compared to a carbohydrate based breakfast. There were no differences in hormone response, subjective appetite, nor lunch *ad libitum* intake between the two protein sources. This indicates that solid, animal-based protein meals and liquid, plant-based meals containing 30 g of protein can be used as strategies for increasing protein intake without influencing subsequent meal calorie intake when consumed at breakfast in healthy young adults and older adults. Future research is needed to confirm acceptability of liquid, plant-based meals and assess long-term adoption in dietary interventions.
